# Factors Affecting the Competence of Nursing Assistants in Taiwan Long-Term Care Institutions

**DOI:** 10.3390/ijerph17249413

**Published:** 2020-12-15

**Authors:** Tsai-Jung Cheng, Yi-Min Hsu, Tung-Han Tsai, Ming-Yu Chen, Shwu-Feng Tsay, Shwn-Huey Shieh

**Affiliations:** 1 Department of Health Services Administration, China Medical University, Taichung 40402, Taiwan; kiki08011015@gmail.com (T.-J.C.); khhoffice@gmail.com (T.-H.T.); nhphoenix@mohw.gov.tw (S.-F.T.); 2 Department of Public Health, China Medical University, Taichung 40402, Taiwan; n4006@mail.cmuh.org.tw; 3 Department of Nursing, China Medical University Hospital, Taichung 40402, Taiwan; 4 Taichung Hospital attached Nursing Home, Head Nurse Ministry of Health and Welfare, Taichung 40343, Taiwan; ninachen196407@yahoo.com.tw; 5 Department of Nursing and Health Care, Ministry of Health and Welfare, Taipei 11558, Taiwan; 6 Department of Nursing, Asia University, Taichung 41354, Taiwan

**Keywords:** care competence, nursing assistants, disabled elderly, long-term care

## Abstract

With the increasing number of people with disabilities caused by an aging global population, the need for long-term care is gradually increasing. Nursing assistants (NAs) are the primary providers of direct care services to older adults with disabilities, whose knowledge, skills, and beliefs affect the quality of care provided. This study aimed to investigate the influential factors affecting NAs’ current competences. A total of 255 NAs’ valid questionnaires were collected from 20 long-term care institutions in Taiwan through convenience sampling. The questionnaire comprised dimensions of demographics and care competence. The study results indicated that NAs had the greatest care competence in the domain of recognition of patient rights (4.64 ± 0.54 points). The multiple regression indicated that age, religion, job category, disability care experience, the receiving of performance bonuses, and the receiving of year-end bonuses significantly affected the level of care competence (*p* < 0.05). With the aforementioned findings, the results of this study serve as references for the government in employing long-term care NAs and developing management policies. Training programs for NAs should be developed to improve the quality of care provided to older adults with disabilities.

## 1. Introduction

An aging population is a serious problem of global concern. According to statistics from the United Nations, 9% of people worldwide were >65 years old in 2019, and this figure is projected to increase to 16% by 2050 [1]. Taiwan’s society became an aged society in 2018, and it is estimated to become a super-aged society by 2026 [2]. Similar to other developed countries, Taiwan’s low total fertility rate has transformed family structures in Taiwan. Consequently, care functions from familial support have declined, and this has, in turn, increased the long-term care (LTC) needs and social burden in Taiwan. Therefore, the promotion of LTC has become an urgent policy matter in Taiwan and many other countries.

LTC types in Europe and North America involve assisted living facilities, skilled nursing facilities, continuing-care retirement communities, medical foster care, and home health care. However, institutional LTC in Taiwan includes LTC institutions, retirement homes, and nursing homes. Nursing assistants (NAs) account for 66%–70% of care manpower but are engaged in 80–90% of care work [3,4,5]. Therefore, NAs are an indispensable source of manpower for LTC institutions. Low wages, lack of good benefits, heavy workload, and lack of promotion channels are all restrictions on the personal and professional development of NAs [6]. In addition, the care competence of NAs is highly correlated with LTC quality [7,8,9,10].

Studies have indicated the importance of professional health care knowledge, efficient interpersonal communication techniques, and moral responsibility in ensuring that the care services rendered to older adults by NAs are of satisfactory quality [11,12]. NAs’ core care competences comprise knowledge on health care, care plan formulation, communication skills, cross-professional team care, safety, professional responsibilities, and ethics [13]. NAs are assistive personnel without formal academic certificates who must undergo training on body mechanics, nutrition, anatomy and physiology, cognitive impairments, mental health, infection control, and personal care skills [14,15]. A recent study indicated that NAs must possess the following care competences for professional and high-quality care: autonomy, daily functioning prevention of health problems, healthy aging and wellbeing, involvement of informal care, collaboration between professionals, and informal care [16]. However, a study indicated that education and training programs are not the primary determinants of NA care quality [17]. The development of leadership skills and services, including personal improvement, civic engagement, communication, and professionalism are also affecting factors [5]. By contrast, LTC institutional culture and LTC feedback to NAs regarding their work performance are the crucial determinants [18]. A recent study done in Australia reported that NAs did not improve in their care competences with experience; however, NAs with more than 5 years of work experience tended to exhibit a more positive work attitude [19].

As indicated in the aforementioned studies, the demand for NAs has been in increasing. NAs are the primary providers of direct care services to older adults with disabilities, and their knowledge, skills, and beliefs affect the quality of care provided. However, the literature on Asia has rarely explored the occupational competences of NAs. Therefore, the present study in Taiwan investigated (1) which critical care competences NAs must possess in providing care to individuals with disabilities and (2) the main factors affecting these care competences of NAs.

## 2. Materials and Methods

### 2.1. Study Design

This cross-sectional study identified prospective participants from 20 assisted living residences, retirement homes, and nursing homes through convenience sampling; these institutions were situated in Northern Taiwan (7 institutions), Central Taiwan (7 institutions), Southern Taiwan (4 institutions), and Taiwan’s outlying islands (2 institutions). Questionnaires were distributed in July 2019 to prospective participants. This study protocol was approved by the Institutional Review Board of China Medical University Hospital, Taiwan (CMUH107-REC2-166), with accreditation by the international association accreditation research protection programs (AAHRPP).

### 2.2. Sample and Setting

Participants had to satisfy the following inclusion criteria: they had to be (a) individuals with NA training qualifications obtained in Taiwan, (b) NAs who had cared for five or more older adults with disabilities, (c) NAs with >6 months of experience in caring for older adults with disabilities, and (d) lucid individuals who could complete the questionnaire and communicate in either Mandarin Chinese or Taiwanese Hokkien. Both Mandarin Chinese and Taiwanese Hokkien are native languages in Taiwan. Thus, there was no need for language translation in the study. In total, 269 NAs completed the questionnaire, and 255 of them provided valid questionnaires, for a response rate of 95%.

### 2.3. Measures

This study used structured questionnaires to collect data. NAs responded to the questionnaires independently. The demographic variables comprised sex, age, monthly salary, bonus, nationality, marital status, level of education, previous disability care experience, religion, education level, college major, nationality, job category, employment type, years of experience, and average hours worked daily. Care competence-related data were collected using the Perceived Caring Ability Assessment Scale of Nurse-Aides in Long-Term Care Facilities developed by Tsai (2013), who approved this study’s use of the scale [20]. This scale comprises six dimensions and 50 items. All items were scored on a 5-point Likert scale, with 1 representing strongly disagree and 5 representing strongly agree. The dimensions (and the number of items in them) are as follows: assistance in daily activities (13), medical professionalism (14), mental and spiritual care (14), professional ethics acknowledgment (4), health education and literacy (3), and recognition of patient rights (2). The Cronbach’s alpha of each dimension was between 0.81 and 0.96. After an expert validity analysis conducted by 10 experts, the content validity index of the scale was noted to be 0.92–0.95.

### 2.4. Statistical Analysis

Descriptive statistics were used to summarize distributions of NAs’ characteristics, including demographics, the types and characteristics of their workplaces, and the score of care competence. The differences between NAs’ demographics, the types and characteristics of their workplaces, and care competences were examined using a t test and analysis of variance (ANOVA). According to the sample size and related experts and scholars in Taiwan, the years of experience were categorized into <1, ≥1 and <3, ≥3 and <5, ≥5 and <10, and ≥10 years. The monthly salary of the study was divided into four groups according to the payroll bracket table of the National Health Insurance Administration, Taiwan. Moreover, after all other relevant variables were controlled for, and multiple regression was conducted to determine the crucial factors affecting NAs’ competences in caring for older adults with disabilities. IBM SPSS Statistics for Windows version 22.0 (IBM Corp., Armonk, NY, USA) was used to conduct statistical analysis. Statistical significance in this study was defined as *p* < 0.05.

## 3. Results

Table 1 presents the data on the participating NAs’ demographic characteristics and overall care competence level. The majority were female (86.3%) and married (60.8%). For the age variable, most participants were in the 31–40 year old group (28.6%), followed by the 41–50 year old group (25.1%). The ≥61 year old group was the smallest (7.5%). For level of education, 78% of the participants graduated from high school or above. Only 29.4% of the participants graduated in a health-related major. For job category, most of the participants worked in nursing homes (56.9%). Almost all of them were full-time workers (95.3%). The proportion of participants with 5 years of experience or longer reached 45.4%. Most of the participants worked an average of 8–12 h daily (76.9%). Only 39.6% of the participants had previous disability care experience. Among the participants, 69.4% had a monthly salary reaching NT$27,001 or above; 65.5% had been receiving work performance bonuses, and 78.8% had been receiving year-end bonuses. In terms of care competence, the participants with an age of 51–60 years, a native-Taiwanese nationality, 5 years of experience or more, previous disability care experience, work performance bonuses, and year-end bonuses exhibited greater care competences. The results reached statistical significance (*p* < 0.05).

Table 2 depicts the importance of NAs’ care competence domains to care for individuals with disabilities. The recognition of patient rights was the most important domain, followed by health education and literacy, professional ethics acknowledgment, assistance in daily activities, medical professionalism, and mental and spiritual care.

Table 3 presents the multiple regression results, which indicate the crucial factors affecting NAs’ competences in caring for older adults with disabilities in each domain. Relative to NAs aged 20–30 years, NAs aged 51–60 years had significantly greater care competence in the domains of assistance in daily activities (β = 0.22, *p* < 0.01), medical professionalism (β = 0.18, *p* = 0.02), and mental and spiritual care (β = 0.25, *p* < 0.01). Furthermore, compared with NAs without religion, NAs with the religion of Christianity/Catholicism (β = 0.26, *p* < 0.001) and Taoism (β = 0.20, *p* < 0.01) had greater care competence in the domain of recognition of patient rights. In the domain of professional ethics acknowledgment, NAs working in assisted living residences (β = 0.23, *p* = 0.04) and adult day-care centers (β = 0.20, *p* = 0.04) exhibited greater care competence compared with NAs working in retirement homes. NAs with ≥5 and <10 years of experience (β = −0.21, *p* = 0.02) and ≥10 years of experience (β = −0.19, *p* = 0.04) exhibited lower care competence.

NAs with previous disability care experience exhibited greater care competence levels in the domains of assistance in daily activities (β = 0.18, *p* < 0.001), medical professionalism (β = 0.18, *p* < 0.001), mental and spiritual care (β = 0.23, *p* < 0.001), and recognition of patient rights (β = 0.16, *p* < 0.01). Relative to those who did, NAs who were not receiving performance bonuses had lower care competence in the domain of professional ethics acknowledgment (β = –0.13, *p* = 0.04), and NAs who were not receiving year-end bonuses had lower health education and literacy (β = –0.18, *p* = 0.03).

## 4. Discussion

With the increasing number of people with disabilities caused by an aging global population, the need for long-term care is gradually increasing. NAs are the primary providers of direct care services to older adults with disabilities, and their knowledge, skills, and beliefs affect the quality of care provided. This study investigated (1) which care competences are crucial for NAs in caring for people with disabilities and (2) the main factors influencing these care competences. The results of the study indicate that NAs’ care competence was greatest in the domain of recognition of patient rights, followed by health education and literacy, but poorest in the domain of mental and spiritual care. The NA’s age, level of education, disability care experience, receiving performance bonuses, and receiving of year-end bonuses were correlated with their care competence.

Among the participating NAs who were taking care of older adults with disabilities, most were married women with a high school education or above. These demographic characteristics are similar to those noted in studies conducted outside of Taiwan [21]. However, this study’s participating NAs were mostly 31–60 years old and were thus older than their overseas counterparts. This is possibly because most of the NAs in Taiwan are those who made a mid-career change from hospital caring to LTC. Compared with hospital caring work, LTC work is more stable [22,23].

NAs had the greatest care competence in recognition of patient rights, followed by health education and literacy, whereas mental and spiritual care was the lowest. This is attributable to the emphasis, by the Taiwanese LTC policies and by assessments of LTC institutions, on 1) care recipients’ perspectives in requirements of older adult care quality and 2) the delaying of disability in care services. However, in Taiwan, patients bear the cost of LTC institutions’ services, which are not covered by public health insurance. The ratio of NAs and care recipients in LTC institutions are on the high side to reduce the cost of long-term care. The daytime care ratio of NAs to care recipients ranges from 1:10 to 1:15, and the night-shift care ratio ranges from 1:25 to 1:30. Considering these labor constraints, the patient rights, daily activities, and health education competence are prioritized to the neglect of mental and spiritual care. Moreover, the education and training of mental and spiritual care among the training programs for NAs in Taiwan should be strengthened. Studies have also suggested that NA education and training should emphasize older adults’ social and health care needs in the domains of autonomy, daily functioning, prevention of health problems, healthy aging, and collaboration with health care professionals [16,24]. The results of this study indicate that there is still room for improvement regarding the training programs of mental and spiritual care for NAs in Taiwan.

The participating NAs who were aged 51–60 years and who had disability care experience tended to have greater care competence in the domains of assistance in daily activities, medical professionalism, and mental and spiritual care. In general, more experienced LTC personnel provide better care [25,26]. A Norwegian study interviewed nurses in a nursing home; it indicated that the hospital admission rate among care recipients was influenced by the nursing home’s human resources, personnel deployment, and workers’ care competence [27]. NA care behavior affects the care recipient’s mental health [28,29,30]. More experienced NAs were found to be more emotionally competent [31]. Compared with their counterparts without care experience, NAs with care experience are more able to provide physiological, psychological, and spiritual care centered on care recipients, thereby improving their care quality. The participating NAs in this study who were receiving performance and year-end bonuses had greater care competence. This result is consistent with those of previous studies demonstrating that incentive policies increase NAs’ work satisfaction and care competence [32].

In Taiwan, there are currently about 30,000 NAs working in retirement centers and nursing home institutions in Taiwan. The nursing care training and qualification requirements of NAs are mostly 90 h (50 h core courses established by the Ministry of Health and Welfare of the Taiwan government, including training on body mechanics, nutrition, anatomy and physiology, cognitive impairments, mental health, infection control, hospice care, and personal care skills, etc.) and a 40 h practice course (10 h of demonstrations and 30 h of clinical practice). Taiwan’s Ministry of Education, following the government’s promotion of its long-term care program 1.0 starting in 2007, has facilitated NA training in colleges and universities. As of 2018, there are about 6530 graduates. However, the salary of NAs in Taiwan is relatively low (32,000 NTD/month or 1150 USD/Month), and institutions have difficulty retaining graduates, with only about 20 to 30% of graduates remaining in the elderly and disability care industry. Of the NAs in Taiwan (about 30,000 in 2019) more than 97% are qualified through the 90 h of training required under the Ministry of Health and Welfare Taiwan (MOHW). NA training courses include lectures, implementation, and clinical practice. Current training courses focus on physical care services. However, NAs simultaneously play the roles of caregiver, companion, and communicator in their provision of comprehensive physical and spiritual care to older adults. The Taiwanese government and related professional organizations should refer to European countries such as Denmark and Finland to nurture NAs or increase the number of theoretical and practical training hours for NAs as done in France, the UK, Australia, and Canada. They can also consider the Netherlands, the UK, and South Korea, where professional grading systems are implemented to enhance the care competence of NAs [33]. In addition to education and training, the professional acknowledgment and welfare of NAs in Taiwan are lower than those in other countries. Therefore, adequate incentives should be provided to increase the care competences of NAs. This study’s limitation lies in its use of convenience sampling, for reasons of limitations in time and resources. Although convenience sampling yields poorly generalizable results, the results are somewhat representative of Taiwan because the participants were from LTC institutions from many parts of Taiwan, specifically, Northern Taiwan, Central Taiwan, Southern Taiwan, and Taiwan’s outlying islands. The results of this study serve as references for the government in the recruitment of NAs for LTC and management policy development. Training programs for NAs should be developed to improve the quality of care provided to older adults with disabilities.

## 5. Conclusions

NA care competence was greatest in the domain of recognition of patient rights, followed by health education and literacy, but poorest in the domain of mental and spiritual care. The NA’s age, level of education, disability care experience, the receiving of performance bonuses, and the receiving of year-end bonuses were correlated with their care competence. The results of this study serve as references for the government in employing long-term care NAs and developing management policies. Training programs for NAs should be developed to improve the quality of care provided to older adults with disabilities. Future research could possibly seek to explore the difference between NAs receiving formal school education and those receiving the MOHW “Nursing Assistant training program” in the competence of disabled care in order to consider adjusting NA education and training content or duration.

## Figures and Tables

**Table 1:** The overall competence level of disability nursing assistants.

Variables	Overall Competence Level (*N* = 255)						
	*N*	%	Score				*p*-Value
			Sum	Mean	±	SD	
Age (year)							0.03
20–30	41	16.1	166.5	4.06	±	0.47	
31–40	73	28.6	287.8	3.94	±	0.49	
41–50	64	25.1	256.7	4.01	±	0.44	
51–60	58	22.7	244.0	4.21	±	0.44	
≥61	19	7.5	77.2	4.06	±	0.43	
Sex							0.07
Male	35	13.7	137.0	3.91	±	0.54	
Female	220	86.3	895.2	4.07	±	0.45	
Marital status							0.76
Married	155	60.8	630.0	4.06	±	0.48	
Single	82	32.2	329.4	4.02	±	0.47	
Divorced/separated	18	7	72.7	4.04	±	0.30	
Religion							0.25
None	59	23.1	233.5	3.96	±	0.43	
Buddhism	76	29.8	309.8	4.08	±	0.42	
Christianity/Catholicism	58	22.7	240.1	4.14	±	0.52	
Taoism	60	23.5	241.1	4.02	±	0.50	
Other	2	0.9	7.7	3.85	±	0.04	
Level of education							0.78
Elementary or lower	12	4.7	48.5	4.04	±	0.34	
Middle school	44	17.3	175.2	3.98	±	0.43	
High school	91	35.7	367.0	4.03	±	0.44	
Trade/Technical college	60	23.5	245.7	4.09	±	0.55	
University or above	48	18.8	195.8	4.08	±	0.45	
Graduated with majors							0.36
Healthcare-related	75	29.4	306.7	4.09	±	0.47	
Else	180	70.6	725.5	4.03	±	0.46	
Nationality							0.03
Native	234	91.8	951.5	4.07	±	0.46	
Non-native	21	8.2	80.7	3.84	±	0.49	
Job category							0.87
Retirement home	18	7.1	72.2	4.01	±	0.48	
Assisted living	49	19.2	199.6	4.07	±	0.42	
Long-term care	11	4.3	44.4	4.04	±	0.45	
Adult day-care	16	6.3	65.0	4.07	±	0.44	
Nursing home	145	56.9	588.5	4.06	±	0.50	
Home care	16	6.2	62.5	3.90	±	0.29	
Employment type							0.06
Full-time	243	95.3	986.5	4.06	±	0.46	
Part-time	12	4.7	45.6	3.80	±	0.44	
Average daily work hours							0.54
Less than 4 h	3	1.2	11.2	3.72	±	0.31	
4–7 h	50	19.6	200.7	4.01	±	0.42	
8–12 h	196	76.9	795.4	4.06	±	0.48	
12 h or more	6	2.3	24.9	4.15	±	0.42	
Years of experience							<0.01
<1 yr	31	12.2	121.5	3.92	±	0.47	
≥1 and <3 yrs	56	22.0	217.0	3.87	±	0.45	
≥3 and <5 yrs	52	20.4	211.3	4.06	±	0.44	
≥5 and <10 yrs	63	24.7	259.7	4.12	±	0.48	
≥10 yrs	53	20.7	222.6	4.20	±	0.43	
Previous disability care experience							<0.001
Yes	101	39.6	421.5	4.17	±	0.44	
No	154	60.4	610.7	3.97	±	0.47	
Monthly salary (NTD)							0.13
22,001–27,000	78	30.6	311.6	3.99	±	0.44	
27,001–32,000	84	32.9	336.8	4.01	±	0.48	
32,001–37,000	76	29.8	315.7	4.15	±	0.46	
≥37,001	17	6.7	68.1	4.00	±	0.49	
Performance bonus							<0.01
Yes	167	65.5	686.3	4.11	±	0.47	
No	88	34.5	345.9	3.93	±	0.43	
Year-end bonus							0.04
Yes	201	78.8	820.0	4.08	±	0.47	
No	54	21.2	212.2	3.93	±	0.44	

**Table 2:** Domains of competency level in disability nursing assistants.

Domain	Mean	SD	Rank
Assistance in daily activities	4.30	0.52	4
Medical professionalism	4.30	0.55	4
Mental and spiritual care	3.91	0.62	6
Professional ethics acknowledgment	4.42	0.75	3
Health education and literacy	4.62	0.52	2
Recognition of patient rights	4.64	0.54	1

**Table 3:** Factors affecting competency level in different domains in disability nursing assistants.

Variables	Assistance in Daily Activities		Medical Professionalism		Mental and Spiritual Care		Professional Ethics Acknowledgment		Health Education and Literacy		Recognition of Patient Rights	
	β	*p*-Value	β	*p*-Value	β	*p*-Value	β	*p*-Value	β	*p*-Value	β	*p*-Value
Age (year)												
20–30 (ref.)												
31–40	0.02	0.77	−0.04	0.66	−0.04	0.64	−0.08	0.30	−0.02	0.78	−0.01	0.87
41–50	0.07	0.39	0.04	0.57	0.02	0.78	−0.08	0.32	0.06	0.42	0.01	0.96
51–60	0.22	<0.01	0.18	0.02	0.25	<0.01	0.08	0.26	0.15	0.06	0.15	0.06
≥61	0.02	0.77	0.01	0.92	0.09	0.17	−0.11	0.11	0.08	0.24	0.06	0.37
Religion												
None (ref.)												
Buddhism	0.07	0.35	0.10	0.19	0.07	0.43	0.01	0.96	0.03	0.72	0.01	0.99
Christianity/Catholicism	0.09	0.24	0.01	0.58	0.04	0.64	0.05	0.48	0.09	0.23	0.26	<0.001
Taoism	0.16	0.05	0.13	0.10	0.15	0.05	0.06	0.43	0.15	0.05	0.20	<0.01
Other	0.13	0.05	0.12	0.06	0.16	<0.01	0.08	0.24	0.08	0.23	0.15	0.02
Level of education												
Elementary or lower (ref.)												
Middle school	0.06	0.58	−0.05	0.67	0.05	0.65	−0.07	0.78	−0.07	0.52	−0.09	0.44
High school	0.05	0.70	−0.03	0.86	0.08	0.55	−0.26	0.24	−0.03	0.81	−0.09	0.50
Trade/Technical college	0.03	0.82	−0.13	0.27	0.00	0.99	0.02	0.93	−0.05	0.66	0.02	0.84
University or above	−0.10	0.45	−0.17	0.18	−0.05	0.72	−0.09	0.69	−0.15	0.24	−0.19	0.14
Graduated with majors												
Healthcare-related (ref.)												
Else	0.04	0.54	0.06	0.35	0.07	0.25	−0.10	0.10	0.02	0.75	0.04	0.48
Nationality												
Native (ref.)												
Non-native	0.02	0.81	0.03	0.64	0.08	0.21	0.04	0.55	0.01	0.94	0.03	0.59
Job category												
Retirement home (ref.)												
Assisted living	0.04	0.71	0.10	0.39	0.13	0.23	0.23	0.04	0.02	0.84	0.10	0.36
Long-term care	−0.05	0.54	−0.06	0.50	−0.01	0.93	0.08	0.36	−0.10	0.25	−0.07	0.44
Adult day-care	0.04	0.70	0.10	0.30	0.01	0.87	0.20	0.04	0.02	0.80	0.09	0.30
Nursing home	0.07	0.59	0.14	0.29	0.03	0.80	0.22	0.09	−0.10	0.43	0.11	0.38
Home care	−0.02	0.81	0.06	0.53	0.10	0.26	0.14	0.11	−0.01	0.91	0.09	0.34
Employment type												
Full-time (ref.)												
Part-time	−0.05	0.43	−0.06	0.38	0.03	0.64	0.03	0.67	−0.03	0.61	−0.02	0.75
Years of experience												
<1 yr (ref.)												
≥1 and <3 yrs	−0.02	0.84	−0.02	0.86	−0.08	0.39	−0.14	0.13	0.10	0.39	−0.08	0.38
≥3 and <5 yrs	0.08	0.40	0.10	0.29	−0.02	0.86	−0.09	0.31	−0.04	0.74	−0.12	0.19
≥5 and <10 yrs	0.03	0.71	0.03	0.76	−0.09	0.36	−0.21	0.02	−0.09	0.45	−0.11	0.25
≥10 yrs	0.11	0.17	0.08	0.35	0.02	0.77	−0.19	0.04	−0.07	0.64	−0.08	0.30
Previous disability care experience												
No (ref.)												
Yes	0.18	<0.001	0.18	<0.001	0.23	<0.001	0.09	0.17	0.12	0.05	0.16	<0.01
Monthly salary (NTD)												
22,001–27,000 (ref.)												
27,001–32,000	0.08	0.33	0.12	0.12	0.05	0.56	−0.04	0.63	−0.06	0.47	−0.03	0.74
32,001–37,000	0.05	0.50	0.09	0.27	−0.02	0.76	−0.10	0.19	−0.03	0.75	0.00	0.98
≥37,001	0.03	0.63	0.02	0.75	−0.01	0.92	−0.02	0.73	−0.04	0.59	−0.01	0.87
Performance bonus												
Yes (ref.)												
No	−0.13	0.19	−0.04	0.66	0.02	0.77	−0.13	0.04	−0.08	0.18	−0.08	0.23
Year-end bonus												
Yes (ref.)												
No	−0.08	0.40	−0.02	0.82	0.02	0.81	−0.16	0.05	−0.18	0.03	−0.11	0.08
